# Supporting women with learning disabilities in infant feeding decisions: A scoping review

**DOI:** 10.1111/mcn.13318

**Published:** 2022-01-28

**Authors:** Clare Johnson, Emma Douglass, Geraldine Lucas, Sally Dowling

**Affiliations:** ^1^ School of Art and Design, Faculty of Arts, Creative Industries and Education University of the West of England Bristol UK; ^2^ School of Health and Social Wellbeing, Faculty of Health and Applied Sciences University of the West of England Bristol UK

**Keywords:** breastfeeding, grey literature, health resources, infant feeding, learning disabilities, review, visual images

## Abstract

Mothers with learning disabilities face many challenges during the perinatal period including preparing for and establishing infant feeding. Evidence shows that women with learning disabilities are less likely to breastfeed than other mothers. A scoping review was undertaken using Arksey and O'Malley's methodology to understand what is known about how women with learning disabilities can be supported to make infant feeding decisions, particularly in relation to the use of appropriate and accessible images. An additional aim was to understand what further research is needed to achieve sustainable improvements to policy and practice in this area. A comprehensive search of fourteen electronic databases was undertaken to look for both published and grey literature. Initial searches, after removal of duplicates, resulted in 467 primary research articles plus 22 items of grey literature. Following a systematic process, three published papers and six items of grey literature were identified which met inclusion and exclusion criteria, five of which were resources. Little is known about the acceptability of existing resources, specifically in relation to the use of visual images. A synthesis of the grey literature and a thematic analysis of published literature was conducted and confirmed that women with learning disabilities need tailored support with infant feeding, including accessible resources and that there is a need for more in‐depth research in this area. There is a high level of agreement about the importance of using easily read visual images within these resources, but little evaluation of the types of imagery used or their aesthetic histories.

## Key messages

- Women with learning disabilities need tailored support with infant feeding, including accessible resources.
- Little is known about the acceptability of existing resources on infant feeding to women with learning disabilities, specifically in relation to the use of visual images.
- We found a small number of existing resources, which are similar in approach but not always easy to access.
- The different disciplinary backgrounds of the researchers enabled us to consider the needs of mothers with learning disabilities alongside the history of visual representations of learning disability.

## 1 INTRODUCTION

Learning disability includes a ‘significant reduced ability to understand new or complex information and to learn new skills, with a reduced ability to cope independently’ (Department of Health, 2001, p. 4). Mothers with learning disabilities face many challenges during the perinatal period including preparing for and establishing infant feeding. Evidence shows that mothers with a learning disability are less likely to breastfeed (Goldare et al., 2015; Guay et al., 2017; Hindmarsh et al., 2015), despite the current World Health Organization and Unicef recommendation that infants be exclusively breastfed for the first 6 months of life (WHO/Unicef, 2003).

The Convention on the Rights of Persons with Disabilities (United Nations, 2006) advocates that in order for persons with a disability to make decisions, mechanisms of support should be available. In line with the Equality Act (2010) healthcare professionals in the UK are required to make reasonable adjustments, which includes the provision of accessible antenatal information. To be accessible, information should be ‘able to be read or received and understood by the individual or group for which it is intended’ (NHS England, 2017, p. 6). However, a study by Porter et al. (2012) identified a paucity of antenatal resources for pregnant women who have learning disabilities, and a systematic review of antenatal care provision for women with learning disabilities (Homeyard et al., 2016) identified that this population often struggle to understand antenatal communication and information, with findings suggesting that midwives lack knowledge in this area and would welcome additional guidance. It appears that these women are not receiving the information they need through the current mechanisms offered.

The Scottish Government articulates the need for quality support, tailored information and family centred approaches throughout pregnancy in their document ‘Supporting Parents with Learning Disabilities in Scotland: Challenges and Opportunities’ (Stewart et al., 2015; Scottish Consortium for Learning Disability, 2015). This document argues that as a central tenet of good practice, better accessibility to quality resources and information can lead to enhanced outcomes for children whose mothers have a learning disability. This includes the fostering of close maternal/infant bonds, which can be achieved through a range of experiences including infant feeding. This approach to infant feeding—whether from the breast or using bottles—is promoted by the Unicef Baby Friendly Initiative and is the basis for the support they offer to maternity, neonatal, health visiting, children's centre services and universities (Unicef, 2021).

It is important that mothers with a learning disability are supported to make informed decisions about infant feeding, to understand the benefits of breastfeeding for the health of women and children (Victora et al., 2016), and to understand that they have choices about how they feed their babies, including safe formula feeding. This scoping review aims to discover what is currently understood about infant feeding choices for women with learning disabilities, paying attention to the use of visual images and where further research is needed to achieve effective and sustainable improvements to policy and practice in this area.

This scoping review is part of a larger piece of work, which aims to understand how women with learning disabilities can be supported to make infant feeding decisions, particularly in relation to the use of appropriate and accessible images. Overall the project has the following aims:

1. What *resources* are available to support this population to make decisions about infant feeding?
2. How do *professionals support* women with learning disabilities to make these decisions?
3. How would women with learning disabilities *like to be supported* to make these decisions?
4. Which *images/media* are most effective in supporting decision‐making in this area?

In addition to the scoping review reported here, the other phases of the project include:

1. Qualitative interviews with health professionals about their experiences of supporting women with learning disabilities in making infant feeding decisions (interviews complete (*n* = 7), analysis in progress, write‐up for publication to follow);
2. Focus groups with women with learning disabilities to show and discuss a range of images of breastfeeding (resources prepared, focus groups currently on hold due to Covid‐related delays in recruitment);
3. A final report synthesising findings from all three phases to make recommendations for practice and/or future research.

## 2 METHODS

### 2.1 Research design

A scoping review is an iterative process in which the relevant literature is identified and synthesised in order that gaps in the existing knowledge base can be identified. Arksey and O'Malley's (2005) scoping review methodology was followed throughout this study. This includes five stages: 1. Identifying the research question. 2. Identifying relevant studies. 3. Study selection. 4. Charting the data. 5. Collating, summarising and reporting the results. We also followed the reporting guidelines in the PRISMA Extension for Scoping Reviews (Tricco et al., 2018), where relevant. Critical appraisal of individual sources of evidence is not usually undertaken as part of scoping review methodology (Arksey & O'Malley, 2005).

### 2.2 Identifying the research question

Our research question, which was developed following a review of the literature and study team discussion, was: *How are women with learning disabilities supported to make infant feeding decisions?* This is also the overall question for our larger project. This review specifically aimed to answer: *What resources are available to support this population to make decisions about infant feeding?* This was underpinned by our specific interest in the use of visual and accessible images.

### 2.3 Identifying relevant studies

A comprehensive search of both scientific and grey literature was undertaken (Arksey & O'Malley, 2005). A search for published literature was conducted in November 2020 with input from two subject librarians from the University of the West of England (UWE), Bristol, Library Service. We searched the electronic databases EBSCO (CINAHL, Medline, AMED, PsycInfo), Embase (Maternity and Soc policy), IBSS, BND, Soc Abs, Scopus, Cochrane and NICE Evidence to access a wide range of health‐related literature as possible. We also searched using Google Scholar, Open Grey and Base to look for grey literature.

The use and appropriateness of search terms evolved during discussions with the librarians assisting us. Our initial searches were too specific and did not result in any useful literature; including terms such as ‘support’ and ‘resources’ led to unmanageable numbers to review. The final search used four concepts, using synonyms for learning disability, infant feeding, mother and health promotion. These were then combined and used in each database (see Table 1). We included all types of research design and excluded papers published more than 10 years ago (i.e., published before November 2010) and those not published in English. Duplicates were removed. Titles and abstracts were retrieved for screening.

**Table 1:** Terms used in database searching

Concept		Concept		Concept		Concept
‘Learning disab*’ **OR** ‘Learning diff*’ **OR** ‘Intellect* impair*’ **OR** ‘Intellect* disab*’ **OR** ‘Develop* disab*’ **OR** ‘Develop* impair*’	**AND**	‘breast fe*’ **OR** ‘breastfe*’ **OR** ‘breast‐fe*’ **OR** lactat* **OR** ‘parent‐infant relations*’ **OR** ‘infant feed*’	**AND**	Mother* **OR** wom*	**AND**	‘Health promot*’ **OR** ‘health educat*’ **OR** ‘public health’ **OR** ‘baby friendly’ **OR** ‘baby‐friendly’

A search of grey literature was initially undertaken independently by all four researchers using Google. The searches used variations on wording which arose from our iterative search terms discussion with the librarians and included: r*esources to support women with learning disabilities with infant feeding, mother and learning disability, women with learning disabilities and infant feeding* and the words *breastfeeding; infant feeding; learning disability; midwifery; midwives and bottlefeeding*. Each researcher looked through the first and subsequent pages generated until the results appeared either very similar to those found or increasingly irrelevant (this happened fairly quickly). We combined our findings and removed duplicates before discussion.

In addition to the database and grey literature searching ED also consulted with some experienced experts and another researcher in this area who was known to us. Arksey and O'Malley (2005) suggest consulting with networks/people working in the area to identify additional grey literature as an optional stage in identifying relevant literature.

These people sent us some additional resources but this did not result in anything that we had not already found through our other search processes.

### 2.4 Study selection

An iterative screening process was followed during a series of meetings to select studies for the review. All four researchers independently read through the titles and abstracts of retrieved literature in line with the pre‐agreed inclusion/exclusion criteria labelling each as either ‘include,’ ‘maybe’ or ‘exclude’. Following the first stage of study selection, the discussed articles/resources were retrieved, and the team agreed to refine the inclusion and exclusion criteria to make the search more specific. This was because some of the retrieved papers/resources were useful contextual information, but not focused on ‘women with a learning disability’ and ‘infant feeding’. The inclusion and exclusion criteria were accordingly amended (see Table 2).

**Table 2:** Final inclusion and exclusion criteria (amendments shown in bold)

Inclusion criteria	Exclusion criteria
Focus is on women/woman/mother(s) who has a learning disability. (Please note that whilst “learning disability” is the term used in England and Wales, the term “Intellectual Disability” or “Intellectual Impairment” is used internationally, so accept all 3 terms).	Focus is on child/children with a learning disability and not mother.
**Focus of paper is on using or developing resources to support infant feeding decisions**. **Or** **Resource includes infant feeding** (Infant feeding in its widest sense, so could be preparation antenatally, bottle feeding, breastfeeding, support, experiences of, etc.) Could be research, antenatal information, or resources.	Focus is on benefits of breastfeeding. (This will be useful for context but not scoping review). Focus is on weaning not early infant feeding **Infant feeding is mentioned as an area of practice requiring further development/accessible resources, rather than focus of paper/resource**.
Any date	
English language	Non‐English language
Any study design/method, policy, strategy, guidance document, material for professionals. Also, leaflets and other resources intended to be used with women with learning disabilities.	

At all stages, researchers erred on the side of caution, marking an article as ‘maybe’ rather than ‘exclude’. The same process was followed for both published and grey literature. During subsequent meetings, all literature on the ‘yes’ and ‘maybe’ list was discussed. Authors were contacted via e‐mail for copies of resources referred to within retrieved articles or via the grey literature search. Full‐text was retrieved for published literature and all four researchers read the papers independently.

### 2.5 Charting the data

Charting the data involves collating key information about the included items in the review, and is similar to data extraction in traditional systematic reviews (Arksey & O'Malley, 2005). In a scoping review, this process can be seen as the first stage in a narrative review (Arksey & O'Malley, 2005). This process was carried out by CJ and GL. There were nine items in total—the two researchers extracted the data for four items each, with the data for one item being extracted by both to ensure parity and consistency of process. The data extracted can be seen in Table 3 (see Results), describing the following:

- title of study
- author(s)
- date of publication
- what form the literature takes (e.g., journal article, report, resource)
- research methods used (if it is a research study)
- extent to which it addresses infant feeding (including breastfeeding and formula feeding)
- if it considers best practices or principles

**Table 3:** Charting the data from included papers and grey literature

Charting the published and grey literature (February 2021)											
Title	Authors	Date	What form?	Research methods (if it's a research study)	Infant feeding/formula feeding/breastfeeding?	Is it about best practices or principles?	Is it an example of an actual resource? If so, what?	If it's a resource, is it: text, easy read, images (what type), other?	If it's a resource, how easy is it to access (e.g., do you have to contact anyone)?	If it's not a resource, what are the key messages or findings?	Data charted by CJ or GL
**Published literature**											
Mothering with an Intellectual Disability: a Phenomenological Exploration of Making Infant‐Feeding Decisions	Guay, A., Aunos, M., & Collin‐Vezina, D.	2017	Journal article published in *Journal of Applied Research in Intellectual Disabilities*. Article is about the experiences of mothers with learning disabilities (LDs) in making and carrying out infant‐feeding decisions.	Interpretative phenomenological analysis; semi‐structured interviews.	Concerns low rates of breastfeeding amongst women with LDs and the first‐hand experiences of four participants with breast/formula feeding.	Discussion aims to inform best practice and recommends further research.	No	N/A	N/A	Importance of the pre‐natal period for educating women about infant feeding. This was when the women interviewed were feeling most empowered as mothers. Health professionals working with women with LDs need to get to know them during the pre‐natal period to understand what motivates their feeding decisions and adjust their educational approach accordingly. Mothers reported different learning needs for breastfeeding (modelling preferred over text or images) and formula (written material sufficed). Small study (four participants) so not generalisable.	CJ
‘We both just wanted to be normal parents’: a qualitative study of the experience of maternity care for women with learning disability	Malouf, R., McLeish, J., Ryan, S., Gray, R., & Redshaw, M.	2017	Journal article published in *BMJ Open*. Studies the lived experiences of pregnancy, childbirth, pre‐natal and post‐natal care and support needs for women with LDs.	Interpretative phenomenological analysis; semi‐structured interviews.	Feeding is discussed as it relates to the experiences of the participants, for example, misunderstanding info about establishing breastfeeding.	Discussion of best practice for health professionals working with women with LDs.	No	N/A	N/A	Four themes identified from interview data: ‘I hate being treated differently,’ ‘I find it harder to understand than other people,’ ‘We've had to prove ourselves’ and ‘Make sure you've got very good support around you’. Findings include the need for health professionals to make reasonable adjustments including adapting to individual learning and communication needs and offering clear explanations of each aspect of care. There are also findings in respect of mothers who are subject to social care assessment.	CJ
To what extent are midwives adapting antenatal information for pregnant women with intellectual disabilities? a survey of NHS trusts in England	Homeyard, C. E., & Patelarou, E.	2018	Journal article published in *Public Health*. The article tried to identify what antenatal information is provided to women who have LDs across different trusts and local supervising authorities.	Mixed methods: focus group with midwives and policymakers, survey questionnaire, interviews with women who have LDs.	General review of antenatal resources, and whether standard information is being disseminated. Breastfeeding adapted resource one of the top three resources available, but only accessible in 16.9% of trusts.	Standard antenatal resources and their availability to women who have LDs.	No	N/A	N/A	Accessible formats not available for women with LDs. Lack of reasonable adjustments. National NICE Guidance recommends accessible information. Lack of post‐registration education on LDs. Lack of resources is detrimental to women's knowledge of pregnancy.	GL
**Grey literature**											
Pregnancy Support Pack	NHS Fife, Porter, E., Kidd, G., Murray, N., Uytman, C., Spink, A., & Anderson, B.	2012	CD‐ROM and journal article in the *British Journal of Learning Disabilities*	Development of antenatal resources for each part of the antenatal care spectrum for parents with LDs. Resources were evaluated through use of structured interviews for women who have LDs and semi‐structured interviews for midwives. Questionnaire was used before dissemination of the resources to elicit medics, midwives and community midwives' knowledge in regard to LDs and communication strategies.	Breastfeeding and formula feeding including topics such as preparing bottles, breastfeeding positions, milk storage and breastfeeding twins.	Aims to inform best practice in the production of antenatal resources for women with LDs.	A CD‐ROM resource	Easy read, uses Picture Communication Symbols	Emailed author to gain access	Acceptability to health professionals and mothers evaluated in a subsequent research paper. Antenatal resources, specifically those that had pictures with some text were deemed as helpful. Women could understand important pregnancy‐related information that would otherwise be difficult to comprehend. Information is accessible. Clarifies support available. Supported staff with information giving and obtaining informed consent. Addresses power imbalances.	GL
Inclusive support for parents with a learning disability	MENCAP in partnership with NCT and ACT	2011	Report on a project funded by the Department of Health. The main aim of the project was to train health professionals working with parents during pregnancy and early parenthood, to be more effective in assisting those with an LD.	Review of current resources, steering group meetings with parents with an LD, survey of midwives about their knowledge of the needs of people with LDs, development of 1‐day training package delivered to 135 participants, evaluation.	Formula and breastfeeding discussed in parents steering group, including tables about their perceptions of both breastfeeding and formula feeding.	Aimed at improving best practice.	No	N/A	N/A	The report makes a number of recommendations including: more widespread training, training as part of the undergraduate curriculum, coordinated resources, joint working, link workers, electronic database of resources, inclusive antenatal and parenting classes for parents with an LD, further research into incidences of breastfeeding in mothers with an LD. Some useful findings from the parents' steering group about existing ante‐natal and post‐natal leaflets and DVDs (point 6.4).	CJ
All About Breastfeeding	Public Health Wales/Easy Read Wales	2021	Resource for new mothers in North Wales.	N/A	Breastfeeding	N/A	It is a resource to help women with LDs learn how to breastfeed and to understand the benefits.	Easy read	Booklet available in hard copy (English and Welsh) or on the Betsi Cadwaladr University Health Board website (https://bcuhb.nhs.wales/health-advice/families-and-early-years/breastfeeding/breastfeeding-booklet-and-other-helpful-information/).	N/A	CJ
CHANGE resources	CHANGE	2000 for the resources on parenting	Website with links to resources including three on parenting: my pregnancy my choice, you and your baby book 0–1, and you and your little child 1–5.	Work is informed by the expertise of people with LDs.	Unknown	Best practice is accessible communication.	Yes: my pregnancy my choice, you and your baby book 0–1, and You and your little child 1–5.	Easy read books	The links to the pregnancy‐related easy read books are not working so they have been requested via email	N/A	CJ and GL
Best Beginings. org.uk/parents‐with‐learning‐disabilities	Best Beginnings	2021	Website. Organisation that works to empower parents and aid children's long‐term development.	Information resource	Parents can download a buddy app that includes information on breastfeeding.	App not reviewed (not LD specific), but website provides information on what is an LD, communication issues, what can enhance antenatal care, potential issues in pregnancy and parenting.	Website pages	Text is quite detailed, but placed under headings. Links to other resources are provided.	Pages on parenting with LDs accessible using the search facility on the Best Beginnings home page.	Suggestions on what can enhance antenatal care for a woman with LDs, communication issues, what is an LD? Issues in pregnancy and parenting, for example, rate of low birthweight.	GL
University of Bristol, Working Together for Parents Network (WTPN) http://www.bristol.ac.uk/sps/wtpn/for/parents	University of Bristol	(n.d.)	Resources for parents who have LDs. Pregnancy and Me: from bump to baby includes making a choice about infant feeding. Links to North Wales ‘All About Breastfeeding’, diagrams and text. Other resources about being good parents, accessing support, and helping parents with LDs to speak up. Under resources: DVD story of a mother with LDs and her experience, practice success stories, policy, WTPN Good Practice Guidance on working with parents with an LD: not being applied in practice, contravenes UN convention for children and human rights, covers safeguarding, accessible information, applies to professional staff.	Resources for parents/staff	Pregnancy and me: from bump to baby encourages consideration of infant feeding method and seeking advice from midwives.	Principles that can be enacted in practical daily life for women with LDs.	Website, leaflets/book	Pictures/links to support networks.	Accessible via web link, but have to click on other links to access documents	Pregnancy: from bump to baby ‐ preparation through journey of pregnancy, method of feeding, asks the client to consider this. Other leaflets include Right support for parents, Being good parents, Helping parents to speak up. Covers themes such as advocacy, support, safeguarding and court issues, being judged as parents.	GL

For resources, data were also extracted about the form the resource takes (e.g., text, easy read and images) and accessibility (how easy it is to access the resource as opposed to accessibility of content for women with learning disabilities). For journal articles and reports, the key messages or findings were also extracted.

### 2.6 Collating, summarising and reporting the results

We conducted a synthesis of the grey literature which involved describing and critiquing the resources found, alongside a thematic analysis of published literature (Thomas & Harden, 2008). These are presented below (Section 3). The data were analysed separately because of the varied nature of the grey literature items and because all published literature was primary research. The synthesis of grey literature was conducted by CJ and GL. The different disciplinary backgrounds of these researchers (visual culture and midwifery) enabled us, through their different areas of expertise, to consider both visual language and information accuracy across the material.

The thematic analysis was conducted by ED and SD. We first copied the ‘results’ text from all three papers into Word documents. Thomas and Harden's (2008) three‐stage approach was used to synthesise this data, which involved line‐by‐line coding of the sections from each paper by ED. Line‐by‐line coding led to the naming of descriptive themes before analytical themes were identified.

### 2.7 Benefits of interdisciplinarity

The study brings together four researchers from different professional backgrounds including visual culture, learning disabilities nursing, midwifery and public health. New interdisciplinary approaches, which pay attention to the ways in which women with learning disabilities identify (or not) with maternal femininity, have the potential to aid communication thus benefiting this community. The project is interdisciplinary because it *integrates* methodological approaches and historical traditions drawn from the different disciplinary backgrounds of the researchers. This differs from a cross‐disciplinary approach in which these disciplinary perspectives are juxtaposed, sitting side by side to achieve the research. It is the integration of social science research methods, clinical experience and attentiveness to visuality that enables a new understanding of the issue of infant feeding choices for women with learning disabilities. This combination of disciplinary backgrounds has encouraged a reflexive approach in which we each recognise our own situatedness within divergent critical discourses and question our own assumptions.

## 3 RESULTS

### 3.1 Characteristics of included material

**Figure 1:**
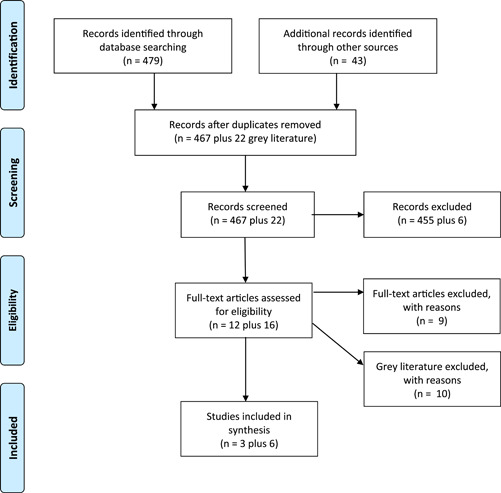
Prisma flow diagram showing search process and article inclusion

After excluding duplicates, 467 published articles and 22 items of grey literature were screened and 9—3 published studies and 6 items of grey literature—were selected for the review. Figure 1 shows the process of searching, inclusion, exclusion and selection. Table 3 gives details of all included items.

Of the six items of grey literature included in this review, five are resources (Public Health Wales, 2021; CHANGE, 2000; NHS Fife and Porter et al., 2012; University of Bristol, Working Together for Parents Network (n.d.); and Best Beginnings, 2021) and one is a report for MENCAP (Leaviss et al., 2011). Leaviss et al. and CHANGE were sources of information that were cited or linked to in other resources, for example, Best Beginnings. All items originate in the UK.

Three published research articles were selected for the review. Guay et al. (2017) interviewed four women with learning disabilities in Canada specifically asking participants about their experiences of infant feeding. Malouf et al. (2017), a UK study, interviewed nine women with learning disabilities exploring their experiences of pregnancy, childbirth and post‐natal care. The third paper (Homeyard & Patelarou, 2018) was a survey of accessible antenatal maternity resources in NHS Trusts in England for women with learning disabilities. Only one of the three studies retrieved was therefore specifically focused on infant‐feeding, however, all were included as they met our inclusion criteria (see Table 2) and incorporated valuable material on support for infant feeding decision‐making. Much of the information from the Malouf et al. study pertains to general maternity experiences, whilst the Homeyard and Patelarou study explores a range of antenatal information.

### 3.2 Overview/synthesis of grey literature

Three of the resources reviewed are ‘easy read’ (Public Health Wales, 2021; CHANGE, 2000; NHS Fife and Porter et al., 2012), which is a format designed to make information accessible using limited text and descriptive visual images. The Public Health Wales resource, ‘All About Breastfeeding: for new mothers in North Wales’ (https://bcuhb.nhs.wales/health-advice/families-and-early-years/breastfeeding/breasfeeding-files/all-about-breastfeeding-easy-read-final-pdf/) aims to help women with learning disabilities learn how to breastfeed and to understand the benefits of breastfeeding. It uses a careful interplay of text and pictures with more complex words highlighted in blue. There is an additional section that provides further context and explanation on these more difficult terminologies. There is a section on how to undertake hand expression, how to breastfeed and, most importantly, where women can access support should they experience any difficulties.

The easy read format is also used by CHANGE, which is a human rights organisation led by people with learning disabilities with a focus on accessibility and equality. CHANGE work alongside people who have lived experience of learning disabilities to coproduce bespoke easy read resources. We were able to review a briefing paper about resources for parents with learning disabilities but were initially unable to access the resources themselves. Eventually, the retail facility of the CHANGE website became functional and we were able to purchase and review the easy read resources. These are substantial ring binders, which contain a wealth of information about pregnancy, birth and parenting approved by UNICEF UK Baby Friendly Initiative. The amount of information is potentially overwhelming. However, the information is divided into sections and includes blank pages at the end of each section for the user to write notes. The resources retail at £45 each, which puts them out of reach for many families. A resource portfolio is available on the CHANGE website, which details publications on pregnancy, caring for 0–1 and 1–5 year‐olds (https://www.changepeople.org/). In Scotland, these books are given free to every parent who has a learning disability, but they are not freely available online.

NHS Fife and Porter et al. also use easy read in their Pregnancy Support Pack, which is an accessible resource designed to address a typical antenatal care pathway for women with learning disabilities. The information on infant feeding includes sterilising and preparing bottles, milk storage and breastfeeding positions. The pack uses Picture Communication Symbols, which is a set of colour and black and white drawings originally developed by Mayer‐Johnson for use in augmentative and alternative communication systems (Mayer‐Johnson, 1999). These symbols are used alongside photographs and line drawings supplied by CHANGE. The Pregnancy Support Pack was developed by the Speech and Language Therapy Department within NHS Fife and its acceptability to health professionals and mothers evaluated in a subsequent research paper (Porter et al., 2012).

Our scoping review includes easy read resources (Public Health Wales and CHANGE), which aim to be inclusive and tackle the communication issues identified in both grey and published literature, for example, difficulties understanding pregnancy information, reluctance to attend antenatal classes, consequent lack of preparedness for labour and birth. Easy read is the accepted format for communicating information to people with learning disabilities, but we did not find any critical evaluation of easy read information being used for infant feeding across either the grey or published academic literature.

Public Health Wales' ‘All About Breastfeeding’ is a useful example of the easy read format. It is divided into three sections: ‘why breastfeeding is best for your baby and for you,’ ‘how to breastfeed and express milk,’ and ‘where to get help and support’. The visuals include both photographs and illustrations, often used in combination on the same page. Layout is uniform with a column of images on the left‐hand side of each page and text in short sentences using a large sans‐serif font on the right‐hand side. There is minimal extraneous page furniture other than a small number of boxouts to comment, for example, on formula feeding and cluster feeding. Photos are used to depict people (e.g., mothers, babies and midwives), social situations (e.g., appointments), and for instruction on how to breastfeed including positions. There is no uniformity of photographic type, style or point of view: some photographs are colour, some black and white, some appear with illustrative elements over them. A combination of illustrative styles is used including line drawings and cartoon imagery. The photographic imagery includes close‐ups of breastfeeding infants, which are potentially more challenging for viewers who may not be comfortable with realistic representations of women's unclothed bodies. Not all of the bodies represented are white, but the breastfeeding close‐ups depict white bodies and are similar to those found in formula advertising. Attention has been paid to the text to ensure it is consistently clear and paired back. However, this level of consistency has not been extended to the imagery.

Further support for professionals is provided by the University of Bristol's, Working Together for Parents Network (n.d.), which extends to England, Wales and Scotland. This network supports professionals who provide care for parents who have learning disabilities. Their website includes resources for parents who have learning disabilities, resources for professionals, information about regional groups and policy guidance.

The resource included in this review with the widest reach is the Best Beginnings (2021). This organisation works on a national and global scale to enhance the outcomes for infants. Their resources aim to support this process from the conception period onwards. The section on their website titled ‘Parents with learning disabilities’ (https://www.bestbeginnings.org.uk/parents-with-learning-disabilities) includes some of the challenges that women might experience in their pregnancy and beyond. Other organisations such as MENCAP and CHANGE are listed as the go to resources if additional support is needed. There is a link to the Baby Buddy app, which is not specific to women with learning disabilities, but considered ‘the perfect tool for mothers with learning disabilities’ (Best Beginnings, 2021). The web page is primarily informative, focussing on issues in pregnancy, issues for antenatal care and education, issues for parenting and issues for communication. There are links to 13 short videos on breastfeeding covering topics such as the first feed, overcoming challenges and expressing.

In addition to resources, our review included a substantial report on a project called Inclusive Support for Parents with a Learning Disability (Leaviss et al., 2011), which was carried out by MENCAP and funded by the UK Department of Health. The main aim of the project was to train health professionals working with parents during pregnancy and early parenthood to be more effective in assisting those with a learning disability. The report makes a number of recommendations including training as part of the undergraduate curriculum, coordinated resources, link workers, inclusive antenatal and parenting classes for parents with a learning disability, and further research into incidences of breastfeeding in mothers with a learning disability.

Across the grey literature, there is an uneven relationship between inclusivity and accessibility of the resource. All of the materials advocate inclusive practices that better serve people with learning disabilities. However, there is variation in the ease with which resources can be accessed with some, but not all, readily available free of charge. It is unclear how frequently these resources are used as well as if they are acceptable and useful to women with learning disabilities.

The grey literature is highly cross‐referential, with particular organisations such as MENCAP and CHANGE emerging as touchstones for information about parenting with a learning disability. Alongside the navigational logic of online resources, this cross‐referencing between key organisations creates a sense of shared messages and a consensus about the need for particular kinds of communication focused on clarity and ease of legibility.

### 3.3 Thematic synthesis of published literature

The following themes were identified across the three studies included in the review: *Support is crucial; Accessibility of information; and Normality/decision‐making is important*.

#### 3.3.1 Support is crucial

Informal and formal support mechanisms are required to support women to make infant‐feeding decisions. Positive and negative experiences of receiving support were reported in the studies by Guay, Aunos and Collin‐Vezina and Malouf et al. Formal support can be through mechanisms including ante‐natal classes and conversations with healthcare professionals such as midwives and doctors, whilst informal support is likely to come from family members and friends.

Participants in the studies by Malouf et al. and Guay, Aunos and Collin‐Vezina spoke about difficulties and challenges with breastfeeding. Wider research suggests women who face breastfeeding challenges have often received poor support and are likely to make decisions about feeding their babies that are not based on good information, with women often feeling they are getting it ‘wrong’ (Thorpe et al., 2020; Trickey & Newburn, 2014). This makes support crucial in supporting women to make infant‐feeding decisions both in the pre and post‐natal periods.

#### 3.3.2 Accessibility of information

There was recognition that a variety of different approaches to the provision of information and infant‐feeding resources are required. Some women found generic information accessible, whilst others preferred easy read. The study by Homeyard and Patelarou (2018) found that whilst 92.7% of NHS Trusts surveyed (74 of 139 Trusts contacted completed the survey) had generic information on breastfeeding, only 16.9% had breastfeeding information in an accessible format. Guay, Aunos and Collin‐Vezina found the pre‐natal period was crucial for making infant feeding decisions, and for providing accessible, tailored information about infant feeding. Some women in the Malouf et al. study found the Internet useful for finding information, with NHS Choices identified as being helpful for some. Videos were identified as being potentially useful (Malouf et al., 2017), but both Guay, Aunos and Collin‐Vezina and Malouf et al., found that many women wanted health professionals to give practical demonstrations:

*“‘I just wanted them to show me, but they would explain it to me and explain it to me and they would show me paper… and I'm like, I don't want you to explain it’ (Paula).”* (Guay et al., 2017, p. 517).

The importance of healthcare professionals checking understanding is important, as examples are given where women have not necessarily understood the information provided by healthcare staff. For one woman, this misunderstanding resulted in them exclusively bottle‐feeding:

*“I said I wanted to do both…I got told that it would mess up the baby's head really… So I just went on bottle‐feeding her*. (Morgan).” (Malouf et al., 2017, p. 5).

#### 3.3.3 Normality – Decision‐making is important

Women with learning disabilities want to make their own decisions, with some participants demonstrating having made an informed decision to breastfeed before the baby was born:

*“‘No, I decided that on my own but I tried to learn as much as I can about it before I gave birth*’ (Paula).” (Guay et al., 2017, p. 516).

Understanding the benefits of breastfeeding appeared to have informed infant‐feeding decision‐making in the Guay, Aunos and Collin‐Vezina study. Participants talked about the importance of personal choice and normality. Although, the findings suggest that it might be difficult to know what ‘normality’ looks like. For example, in the Malouf et al. study, what was interpreted as too much questioning from healthcare professionals by one participant might have been the same level of questioning as used with every woman.

### 3.4 Summary of material reviewed

The published literature suggests that women with learning disabilities need tailored support with infant feeding, including accessible resources. Only 16.9% of NHS Trusts surveyed had accessible information about infant feeding (Homeyard & Patelarou, 2018), identifying a gap in the provision of accessible information available. The grey literature retrieved suggests a uniformity in the resources identified, with a focus on ‘easy read,’ whereas the study by Malouf et al. suggests that other sources of information might be useful, such as videos. What is missing in most instances is an evaluation of the acceptability of the resources available to women with learning disabilities.

## 4 DISCUSSION

### 4.1 The use of visual material and historical context

Amongst the published and grey literature there is a high level of agreement about the importance of using visuals to communicate information about pregnancy and birth to women with learning disabilities, including infant feeding practices. This is understood as a primary means of achieving ‘accessible’ resources, defined as those that are highly visual with minimal text written in plain English (Best Beginnings, 2021; CHANGE, 2000). Best Beginnings, for example, speaks of the importance of presenting information ‘in a format learning disabled people can understand and relate to’ (Best Beginnings, 2021). With exception of the NHS Fife resource, and subsequent evaluation (Porter et al., 2012), the grey literature does not, however, include any discussion of how relatable resources might be achieved or what it means for this community to identify with specific visual languages beyond a list of sensible requirements such as few words on a page, clear ordering of information and pictures containing only one item at a time (Best Beginnings). Homeyard and Patelarou (2018) identified 16.9% of NHS Trusts had breastfeeding information in an accessible format, however, details of what the accessible format was, or how it was received by women with learning disabilities are not known.

Within the literature reviewed there is no discussion of the type of visuals used, their aesthetic histories (e.g., tropes familiar from other visual forms such as charity advertising), photographic codes (e.g., genre, camera angle and composition), ideological implications, or the contexts in which they were made and received.

This is significant given the highly problematic history of representations of people with learning disabilities and something that our inter‐disciplinary approach, which combines learning disability nursing, midwifery, public health and visual culture, seeks to address. Historically, people with learning disabilities have either been absent from visual representation or depicted through othering discourses such as carnival (e.g., late 19th century freak shows) or pity (e.g., charity advertisements). Socially deviant roles—such as outcast, menace, perpetual child or economic drain—pervade this visual rhetoric. Indeed, the visual representation of people with disabilities, including learning disabilities, has historically worked to construct and perpetuate difference to secure a sense of coherence for the viewer. This works to allay the social anxiety caused by what the cultural theorist Margrit Shildrick calls the ‘horror of indeterminacy,’ the fear that the monstrous other may not be so different after all. In this sense, the othering of people with learning disabilities serves a distressing cultural function by shoring up the fantasy of lifelong able‐bodied‐ness (Shildrick, 2002, p. 45).

There have been attempts to reposition the representation of this community in ways that challenge their exclusion from dominant visual languages, but these are few and far between. A notable example is a series of photographs for MENCAP's ‘Here I Am’ campaign by British fashion photographer Rankin (MENCAP, 2016). The campaign aims to tackle the treatment of people with learning disabilities and in response Rankin produced high‐contrast, black and white studio portraits consistent in style with his celebrity photographs of actors and musicians. These portraits work towards integration rather than otherness, but within the differently objectifying discourse of fashion photography. Integration into the visual apparatus of popular culture is, however, fraught with complications. The modelling and acting agency Zebedee, for example, describe themselves as a ‘specialist talent agency,’ which aims to increase the representation of people with disabilities, alternative appearances or who are trans/nonbinary (Zebedee Management, n.d.). As an agency, Zebedee finds paid work as models, performers and actors for many people with learning disabilities who are on their books. However, a scroll through their directory of models reveals an uncomfortable connection with a visual history of fetishization and objectification of the disabled body.

Historically, mothers with learning disabilities are doubly excluded from representation. They are consigned to bodily immanence as a result of their disabilities, understood as unable to ‘transcend’ their bodies to become fully fledged subjects, and positioned outside dominant representations of maternal femininity, which are almost exclusively able‐bodied. There are challenges to this in relation to physical disability—notably Marc Quinn's sculpture *Alison Lapper Pregnant*, which occupied the fourth plinth at Trafalgar Square in London in 2005—but positive maternal representations of women with learning disabilities remain almost non‐existent within art or popular culture. It is widely understood that seeing positive images of the group to which you belong represented in popular culture enables identification and aspiration (Hall, 1997), but what constitutes a positive image of maternal femininity, and infant feeding in particular, needs further discussion in the context of learning disability. In short, the historical context within which the grey literature advocates the use of visuals in learning disability resources raises the stakes. The field of representation is not neutral, but inscribed with problematic representations of inability, deficiency and infantilisation.

There is a need to put women with learning disabilities at the centre of the discussion about resources, to hear their voices so that we can understand which image types improve legibility and understanding. However, we found no evaluation of the easy read format in relation to infant feeding information. Throughout the grey literature reviewed there is a strong sense of advocacy on behalf of this community, but little information about their (audio) visual tastes and preferences. However, there is a reference to the coproduction of resources in materials produced by CHANGE. Across the literature, women with learning disabilities are addressed as users and occasionally co‐producers, but not as visually literate consumers of materials and resources.

### 4.2 Strengths and limitations

The strength of this review is that, as far as we are aware, it is the first to focus on this important area. It has been undertaken using systematic methods. This study has some limitations. Despite considerable effort, some materials were difficult to access. However, when we were eventually able to access these resources they confirmed our findings about the widespread use of ‘easy read’. Two of the published articles were not focussed on infant feeding decisions specifically but were looking at pregnancy more generally. The research was conducted during the Covid‐19 pandemic, which meant that all aspects of the screening process were conducted virtually. This proved a limitation in terms of ease of process, but not of rigour. Finally, we are mindful that none of the researchers in our team has a learning disability, which is an issue for inclusivity and limits our understanding of the impact of existing resources on the community they are targeting. We are considering the feasibility of establishing a steering group including people with learning disabilities for the later phases of this project.

## 5 CONCLUSION

This scoping review reveals the paucity of existing research into the resources available to support women with learning disabilities to make infant feeding decisions. It highlights the need for user‐centred research into the use of visual materials in these resources. Our recommendation is that further studies in this area put the voices of women with learning disabilities, as well as the health professionals who support them, at the heart of the research.

## CONFLICT OF INTERESTS

The authors declare that there are no conflict of interests.

## AUTHOR CONTRIBUTIONS

The work was jointly conceived in discussion with all authors. Searches were carried out with the assistance of librarians from UWE, Bristol Library Service. All authors contributed to the search, screening and study selection process. CJ and GL charted the data from the selected studies and carried out the synthesis of the grey literature resources. ED and SD carried out thematic analysis of the published studies. CJ and ED wrote the first draft of the article; SD edited the final version and all contributed to subsequent reviews. All authors approved the final version of the paper.

## Data Availability

Data sharing is not applicable to this article as no data sets were generated or analysed during the current study.

## References

[mcn13318-bib-0001] Arksey, H. , & O'Malley, L. (2005). Scoping studies: Towards a methodological framework. International Journal of Social Research Methodology, 8, 19–32. 10.1080/1364557032000119616

[mcn13318-bib-0002] Best Beginnings . (2021). *Parents with learning disabilities [online]*. https://www.bestbeginnings.org.uk/parents-with-learning-disabilities

[mcn13318-bib-0003] CHANGE . (2000). https://www.changepeople.org

[mcn13318-bib-0004] Department of Health . (2001). *Valuing people: A new strategy for learning disability for the 21st century [online]*. https://www.gov.uk/government/publications/valuing-people-a-new-strategy-for-learning-disability-for-the-21st-century PMC1314122 11677700

[mcn13318-bib-0005] Equality Act . (2010). *[online] Chapter 15 [2010]* Legislation.gov.uk. http://www.legislation.gov.uk/ukpga/2010/15/pdfs/ukpga_20100015_en.pdf

[mcn13318-bib-0006] Goldacre, A. D. , Gray, R. , & Goldacre, M. J. (2015). Childbirth in women with intellectual disability: Characteristics of their pregnancies and outcomes in an archived epidemiological dataset. Journal of Intellectual Disability Research, 59, 653–663. 10.1111/jir.12169 25331275

[mcn13318-bib-0007] Guay, A. , Aunos, M. , & Collin‐Vezina, D. (2017). Mothering with an intellectual disability: A phenomenological exploration of making infant‐feeding decisions. Journal of Applied Research in Intellectual Disabilities, 30, 511–520. 10.1111/jar.12298 27878910

[mcn13318-bib-0008] Hall, S. (Ed.). (1997). Representation: Cultural representations and signifying practices. Sage Publications.

[mcn13318-bib-0009] Hindmarsh, G. , Llewellyn, G. , & Emerson, E. (2015). Mothers with intellectual impairment and their 9‐month‐old infants. Journal of Intellectual Disability Research, 59, 541–550. 10.1111/jir.12159 25208604

[mcn13318-bib-0010] Homeyard, C. , Montgomery, E. , Chinn, D. , & Patelarou, E. (2016). Current evidence on antenatal care provision for women with intellectual disabilities: A systematic review. Midwifery, 32, 45–57. 26518177 10.1016/j.midw.2015.10.002

[mcn13318-bib-0011] Homeyard, C. , & Patelarou, E. (2018). To what extent are midwives adapting antenatal information for pregnant women with intellectual disabilities? A survey of NHS England. Public Health, 158, 25–30. 10.1016/j.puhe.2018.01.034 29533834

[mcn13318-bib-0012] Johnson, R. M. (1999). The picture communication symbols: Combination book. Mayer‐Johnson Company.

[mcn13318-bib-0013] Leaviss, J. , Ewins, W. , & Kitson, D. (2011). *Inclusive support for parents with a learning disability, MENCAP*. https://www.bl.uk/collection-items/inclusive-support-for-parents-with-a-learning-disability

[mcn13318-bib-0014] Malouf, R. , McLeish, J. , Ryan, S. , Gray, R. , & Redshaw, M. (2017). We both just wanted to be normal parents': A qualitative study of experience of maternity care for women with learning disability. British Medical Journal, 7, 1–10. 10.1136/bmjopen-2016-015526 PMC5372071 28341692

[mcn13318-bib-0015] MENCAP . (2016). *Here I am: Rankin photo shoot*. https://www.mencap.org.uk/blog/here-i-am-rankin-photo-shoot

[mcn13318-bib-0016] NHS England . (2017). *Accessible information standard*. NHS England.

[mcn13318-bib-0017] NHS Fife , Porter, E. , Kidd, G. , Murray, N. , Uytman, C. , Spink, A. , & Anderson, B. (2012). *Pregnancy support pack*. Accessed via email communication with author.

[mcn13318-bib-0018] Porter, E. , Kidd, G. , Murray, N. , Uytman, C. , Spink, A. , & Anderson, B. (2012). Developing the pregnancy support pack for people who have a Learning Disability. British Journal of Learning Disabilities, 40, 310–317. 10.1111/j.1468-3156.2011.00713.x

[mcn13318-bib-0019] Public Health Wales . (2021). *All about breastfeeding: For new mothers in North Wales [online]*. https://bcuhb.nhs.wales/health-advice/families-and-early-years/breastfeeding/breasfeeding-files/all-about-breastfeeding-easy-read-final-pdf

[mcn13318-bib-0020] Scottish Consortium for Learning Disability . (2015). Supported parenting. https://www.scld.org.uk/wp-content/uploads/2015/06/Supported_Parenting_web.pdf

[mcn13318-bib-0021] Shildrick, M. (2002). Embodying the monster: Encounters with the vulnerable self. Sage Publications.

[mcn13318-bib-0022] Stewart, A. , MacIntyre, G. , & McGregor, S. (2015). Supporting parents with learning disabilities. In Scotland: Challenges and opportunities. Scoping exercise on behalf of the Scottish Government, keys to life change fund. *Scottish Consortium for learning disability [online]*. https://www.scld.org.uk/wp-content/uploads/2016/11/Parenting-Report-FINAL-14.11.16.pdf#:%7E:text=Good%20Practice%20Guidelines%20for%20Supporting%20Parents%20with%20a,with%20learning%20disabilities%20within%20the%20National%20Parenting%20Strategy

[mcn13318-bib-0023] Thomas, J. , & Harden, A. (2008). Methods for the thematic synthesis of qualitative research in systematic reviews. BMC Medical Research Methodology, 8, 1–10. 10.1186/1471-2288-8-45 18616818 PMC2478656

[mcn13318-bib-0024] Thorpe, K. , Danby, S. , Cromack, C. , & Gallegos, D. (2020). Supporting, failing to support and undermining breastfeeding self‐efficacy: Analysis of helpline calls. Maternal and Child Nutrition, 16, e12919. 10.1111/mcn.12919 32026573 PMC7083474

[mcn13318-bib-0025] Tricco, A. C. , Lillie, E. , Zarin, W. , O'Brien, K. K. , Colquhoun, H. , Levac, D. , Moher, D. , Peters, M. , Horsley, T. , Weeks, L. , Hempel, S. , Akl, E. A. , Chang, C. , McGowan, J. , Stewart, L. , Hartling, L. , Aldcroft, A. , Wilson, M. G. , Garritty, C. , … Straus, S. E. (2018). PRISMA extension for scoping reviews (PRISMA‐ScR): Checklist and explanation. Annals of Internal Medicine, 169, 467–473. 10.7326/M18-0850 30178033

[mcn13318-bib-0026] Trickey, H. , & Newburn, M. (2014). Goals, dilemmas and assumptions in infant feeding education and support. Applying theory of constraints thinking tools to develop new priorities for action. Maternal and Child Nutrition, 10, 72–91. 10.1111/j.1740-8709.2012.00417.x 22712475 PMC6860269

[mcn13318-bib-0027] Unicef . (2021). *About Baby Friendly [online]*. https://www.unicef.org.uk/babyfriendly/about/

[mcn13318-bib-0028] United Nations . (2006). *Convention on the Rights of Persons with Disabilities (CRPD) [online]*. https://www.un.org/development/desa/disabilities/convention-on-the-rights-of-persons-with-disabilities.html 10.1515/9783110208856.203 18348362

[mcn13318-bib-0029] University of Bristol, Working Together for Parents Network. (n.d.). http://www.bristol.ac.uk/sps/wtpn/

[mcn13318-bib-0030] Victora, C. G. , Bahl, R. , Barros, A. J. , França, G. V. , Horton, S. , Krasevec, J. , Sankar, M. J. , Walker, N. , Rollins, N. C. , & Lancet Breastfeeding Series Group (2016). Breastfeeding in the 21st century: Epidemiology, mechanisms, and lifelong effect. Lancet, 387, 475–490. 10.1016/S0140-6736(15)01024-7 26869575

[mcn13318-bib-0031] WHO/Unicef . (2003). Global strategy for infant and young child feeding. World Health Organization.

[mcn13318-bib-0032] Zebedee Management. (n.d.). http://www.zebedeemanagement.co.uk

